# Supplementary material for: Temperature-dependent polar lignification of a seed coat suberin layer promoting dormancy in Arabidopsis thaliana
Source: Proc Natl Acad Sci U S A. 2025 Feb 7;122(6):e2413627122. doi: 10.1073/pnas.2413627122 (PMC11831162; doi:10.1073/pnas.2413627122)
Supplement: Supplementary file 1 — Appendix 01 (PDF) [file pnas.2413627122.sapp.pdf]

Supplementary Information Appendix

Materials and Methods

Plant material and growth conditions

All genotypes used in this study were in the Col-0 background. *myb107-1* (SAIL_242_B04), *myb107-2* (SALK_203615), *myb9-1* (SALK_149765C) and *myb9*-2 (CS463394) were previously described (1, 2). The double mutant *myb107myb9* was generated by crossing *myb107-2* with *myb9-1. gpat57* and *gpat457* were generated by crossing *gpat4-1* (SALK_106893), *gpat5-1* (SALK_018117) and *gpat7-3* (SALK_064514), which were previously described (3-5). *cad4cad5f5h-1f5h-2, lac5x (lac1,3,5,12,16)* and *ref3-3* were as previously described (6-8). The *pGPAT5*::*mCitrine-SYP122*, *pC4H::3xNLS-Venus, pPAL1::3xNLS-Venus, pPAL2::3xNLS-Venus and pPAL4 ::3xNLS-Venus* lines were as previously described (9, 10). Oligonucleotides used for genotyping are listed in Supporting Information S2.

All plants were grown under standard conditions as described (11). Cold was applied upon bolting by transferring plants to a cold chamber (10-13°C, 16 h/8 h day/night photoperiod, light intensity of 120-130 µmol/m^{2}s, humidity of 60-65%). Seed batches were harvested on the same day from plants grown side by side under identical environmental conditions.

Histology

Histology followed the methods of Demonsais et al (12). Fixation was overnight at 4°C in phosphate buffer (pH 7.2) with 4% formaldehyde and 0.25% glutaraldehyde. Dry seeds were punctured at the start, and all samples were vacuum infiltrated. Samples were embedded in 1.5% agarose, dehydrated in ethanol, cleared in Neoclear, and embedded in paraffin. Sections (thickness 8 µm) were cut with a Cut 4050 microtome (MicroTec), placed on SuperFrost slides (Roth), deparaffinized with Neoclear, and rehydrated with water. Stainings were done as detailed below; dyes were diluted in water unless otherwise noted, with rinsing after each step except for Phloroglucinol staining (Phloro). AurO staining: 5 min in 0.001% AurO, 1 min in 0.05% Toluidine Blue for counter staining, mounted in PBS/glycerol (1:1). FY staining: 10 min in 0.01% FY in 100% EtOH, 1 min in 0.2% Aniline Blue for counter staining, mounted in PBS/glycerol (1:1). BF staining: 1 min in 0.05% BF in 50% EtOH, mounted in PBS/glycerol (1:1). Phloro staining: 3 min in 1% Phloro in 92% EtOH, 7 min in 25% HCl, mounted in lactic acid/glycerol (1:1).

Widefield observation

Histological dye samples were examined with a DM6B microscope (Leica) equipped with a DFC9000GT sCMOS camera and a HC PL APO 20x/NA 0.8 PH2 objective. AurO was excited between 426–446 [nm] and emission was collected between 460–500 [nm], FY was excited at 450-490 [nm] and emission was collected at 500-550 [nm] and BF was excited 540-580 [nm] and emission were collected at 592-668 [nm]. Z-stacks of 9 -14 different seeds per condition were performed for quantification purposes. Z-stacks were acquired with Nyquist sampling and images were subsequently enhanced using the THUNDER Instant Computational Clearing (ICC) algorithm (Leica), which is based on a spatial filtering method. Phloroglucinol slides were observed using bright field imaging, a DMC5400 colour camera (Leica) and a PlanApo 40x/NA1.1 water immersion objective.

Confocal microscopy

Confocal microscopy observed fluorescent transgenic lines after staining seeds with 0.1% propidium iodide (PI) for 10 min. Images were acquired using a Zeiss LSM 800 microscope. Excitation for eGFP, mCitrine, and Venus was at 488 nm, with emission collected from 488-570 nm. PI was excited at 561 nm, with emissions collected from 562-700 nm. Imaging parameters included a 42 µm pinhole, 0.8-2% laser intensity, and z steps of 0.64 µm. Each line was analyzed at least 3 times, and 10 independent complemented lines were analyzed.

Transmission Electron Microscopy (TEM)

TEM was performed as described previously (11). Briefly, samples were fixed in 2.5% glutaraldehyde in cacodylate buffer 0.1M, pH7.0 with Tween 0.01% and post-fixed in 1% uranyl acetate; they were then dehydrated and embedded in Epon resin. For Reynolds staining, samples were stained with 2.5% (w/v) uranyl acetate and Reynolds lead citrate. For H_2O_2 treatment, a 10 min treatment with 10% H_2O_2 was performed before the Reynolds staining. For $KMnO_4$ staining, the samples were stained 45 min with 1% of fresh $KMnO_4$ as described in (Kolbeck et al., 2022). Data was collected on a Talos L120C microscope (ThermoFisher Scientific) operating at 120kV, using a Ceta detector. The Maps 3.17 software package (ThermoFisher Scientific) was used to acquire montage maps of sections. Typically grids were initially imaged at 84x for grid overview and subsequently oi1 region was imaged at 5300x (pixel size 2.648 [nm]) for width quantification or 11000x (pixel size 1.3418 [nm]) for electron density

quantification and finally at 22000x (pixel size 0.65598 [nm]) for lamellae pictures. The exposure time used was 1s, the objective lens was at 85-86% and optic intensity at 0.46-0.53%.

Image treatment and analysis

Images were analyzed using the software Fiji (13). Measurements of fluorescence intensity were done the following way. After removing the average background measured outside the seed, a maximal Z-projection was performed. Then, the oi1 cell layer was manually isolated on the picture by removing the other seed parts; the micropylar and chalazal regions were also removed and not taken into account in the quantification. A threshold was applied before measuring the fluorescence intensity so as to take into account only the signal of interest. For TEM images, stitching was used to obtain high-resolution large fields of view for illustrative images and was done using the Fiji MosaicJ plugin (14). For width measurement on TEM pictures, a Fiji macro was written. Briefly, two lines delimitating the outer or inner oi1 cell wall were manually drawn. Then, segments orthogonal to these lines and spaced everything 50 nm were automatically placed between these two lines. The length of these segments was the read-out for the width measurements. Electron density was measured as described (15). Lines perpendicular to the cell wall measured were manually drawn using Fiji and the electron density measured along these lines was normalized with the oi2 columella background. The average of the measurement taken along the line was taken as one measure. Three different seeds with one or two cells per seeds were analyzed.

RNA extraction

RNA was extracted from developing seed of three siliques at the indicated time point. If mentioned, the endosperm-integument was isolated manually by dissection. Total RNA was then isolated as previously described (16).

RT-qPCR

Total RNAs were treated with RQ1 RNase-Free DNase (Promega, Switzerland) and reverse-transcribed using ImpromII reverse transcriptase (Promega) and random primer (Promega) according to the manufacturer’s recommendations. Quantitative RT–PCR was performed using the ABI 7900HT fast real-time PCR system (Applied Biosystems, Switzerland) and Power SYBR Green PCR master mix (Applied Biosystems). Relative transcript levels were calculated using the comparative DCt method

and normalized to the CDKC2 gene transcript levels. Primers used are listed in Supporting Information S2.

RNAseq

Total RNA was isolated from 11 DAP or 13 DAP developing seeds. Each sample was collected form 4 siliques of 3-4 different plants and seeds were manually dissected to isolate endosperm-integuments tissues. Total RNA was extracted as for RT-qPCR. RNA concentrations were measured by Qubit Fluorometric quantification system (Thermo Fisher Scientific, Switzerland). The TruSeq mRNA stranded kit from Illumina was used for the library preparation with 100 ng of total RNA as input. Library molarity and quality were assessed with the Qubit and Tapestation (Agilent Technologies - DNA High sensitivity chip). Libraries were sequenced on a HiSeq 4000 Illumina sequencer for around 40 M of single reads 50. The data discussed in this publication have been deposited in NCBI's Gene Expression Omnibus and are accessible through GEO Series accession number GSE250191 (https://www.ncbi.nlm.nih.gov/geo/query/acc.cgi?acc=GSE250191) (17).

Gene expression profile

The Galaxy plateform (https://usegalaxy.org/) was used for bioinformatics analyzes. The following tools were used: Trim galor and Sickle, HISAT2 and StringTie to clean align and calculate the differential expressed genes, respectively. The cut-off used for the analysis was à $\log_2(FC) \geq \pm 1$ and a adj. p-value ≤ 0.05. The GO terms were generated using DAVID Bioinformatics Resources (https://david.ncifcrf.gov/).

Generation of transgenic plants

For the *pMYB107::MYB107-eGFP* line, a DNA fragment, consisting of 3511 bp DNA upstream of *MYB109* ATG and 1687 bp of genomic *MYB107* sequences lacking the stop codon was inserted in frame with the eGFP sequence into the binary vector pBA002a (18). To generate the line *pMYB9::NLS-3xmVenus*, a 2108bp DNA fragment upstream of *MYB9* ATG was amplified by PCR with pMYB9_fwd and pMYB9_rev primers allowing gibson assembly. Gibson assembly was performed between *pMYB9* PCR product and a modified destination plasmid pFR7m34GW (19) (containing PspOMI and AatII unique restriction sites upstream of *NLS-3XmVenus* previously cloned between the RB and LB region) after digestion. The resulting constructs were

transformed into Arabidopsis (20). Plants from T1 and T2 generation were used for confocal analysis.

Primers used in this study are listed in Supporting Information S2.

Polyester extraction and monomer measurements

The protocol was adapted from a polyester root extraction protocol described previously (10). 25 mg of seeds were grinded using a pestle and mortar and extracted in isopropanol/0.01% butylated hydroxytoluene (BHT). The seed tissue was then delipidized the seed tissue for 7 days in chloroform-methanol (1:1 v:v) with 0.01% BHT (solvent was changed every 24h), under agitation before being dried for 3 days under vacuum. Depolymerization was performed by base catalysis (Li-Beisson et al., 2013). Briefly, dried seed samples were trans-esterified in 2 mL of reaction medium. 20 mL reaction medium was composed of 3 mL methyl acetate, 5 mL of 25% sodium methoxide in dry methanol and 12 mL dry methanol. The equivalents of 5 mg of methyl heptadecanoate and 10 mg of ω-pentadeca-lactone/sample were added as internal standards. After incubation of the samples at 60°C for 2h 3.5 mL dichloromethane, 0.7 mL glacial acetic acid and 1 mL 0.9% NaCl (w/v) /100 mM Tris-HCl (pH 8.0) were added to each sample and subsequently vortexed for 20 s. After centrifugation (1500g for 2 min), the organic phase was collected, washed with 2 mL of 0.9% NaCl, and dried over sodium sulfate. The organic phase was then recovered and concentrated under a stream of nitrogen. The resulting suberin monomer fraction was derivatized with BSTFA/pyridine (1:1) at 70°C for 1 h and injected out of hexane on a HP-5MS column (J&W Scientific) in a gas chromatograph coupled to a mass spectrometer and a flame ionization detector (Agilent 6890N GC Network systems). The temperature cycle of the oven was the following: 2 min at 50°C, increment of 20°C/min to 160°C, of 2°C/min to 250°C and 10°C/min to 310°C, held for 15 min. 3 independent experiments were performed with 4 replicates for each genotype, respectively, and a representative dataset is presented. The amounts of unsubstituted C16 and C18 fatty acids were not evaluated because of their omnipresence in the plant and in the environment.

Monolignol chemical analysis

Seed coat-enriched samples (SCES) were isolated from 100mg of dry seeds in quadruplicate as described (21). SCES were grinded in liquid N_2 followed by three washes in DMSO to remove starch. Next, SCES were transferred to glass vials and exhaustively extracted using first methanol followed by Chloroform. Analytical

thioacidolysis was performed following the standard procedure by Rolando et al., with slight adaptations according to Robinson and Mansfield and Qi et al. (22-24). Briefly, 20 mg of oven-dried, extractive-free CWR /samples* were suspended in 0.2 mL of freshly prepared 10 % ethanethiol (EtSH) 2.5 % boron trifluoride diethyl etherate (BF_3) in dioxane and incubated at 100°C for 4 h. Released lignin monomers were extracted with ethylacetate, derivatized with N,O-Bis(trimethylsilyl)acetamide (BSA), and then subjected to GC-MS analysis on a Agilent 8890 gas chromatograph with a 5977B mass-selective detector and DB-1 column (30 m, 0.32 mm, 0.1-μm film thickness). For better resolution of the complex chromatograms lignin monomers were quantified using selected ion chromatograms based on characteristic fragment ions for diagnostic, β-O-4-linked lignin units; i.e., m/z 239 for p-hydroxyphenyl (H), m/z 269 for giaiacy (G), m/z 299 for syringyl (S), and m/z 327 for caffeyl (C) lignin monomers (25).

Sodium hypochlorite-induced seed coat decolorization assay

50-150 seeds were imbibed with a solution of 1.5-2.5% NaClO (bleach) and agitated at 1200 rpm and 22°C during a time ranging from 15 minutes to 3.5 hours, depending on the conditions analyzed (NaClO concentration and incubation time are indicated in the legends of Fig. 5A and S5D). Seeds were rinsed twice with water before being documented with a stereomicroscope (Leica) equipped with a CCD color camera. Quantification of the percentage of bleaching was done using the software Fiji. A color threshold was used to outline the seeds, and for each seed, another color threshold was used to outline the bleached region; after application of each threshold, a manual correction step was applied to correct errors in the automatic detection (in particular to eliminate reflections at the surface of the seed). For each seed, the total area of the seed and the area of the bleached region were measured; the percentage of bleaching was defined for each seed as the ratio of these two values, and statistics were calculated over the whole population.

Germination assays

Seeds were surface-sterilized for 10 min in 70% ethanol and sown on agar plates (Warm seeds) or on imbibed paper (Cold seeds). The germination test was performed at 20-22°C under 80 μE/m2/s, 16h white light: 8h dark, 70% relative humidity. They were independently repeated at least 3 times with 3 technical replicates of 80-100 seeds each. Germination event was recorded as radicle protrusion.

For the suboptimal germination test of Warm seeds, a FR/R pulse treatment was applied after 2h of seed imbibition. Seeds were irradiated with a FR pulse (3.69 µmol m-2 s-1) for 5 min and subsequently irradiated with a red (R) pulse (14.92 µmol m-2 s-1) for 5 min (Kim et al. 2019). Seeds were then maintained in the dark for 4 days before germination analysis.

Statistics and graphs

Statistics were done on R. The data homoscedasticity was tested using the Fligner test. If the test was accepted (p-value > 0.05), the ANOVA (parametric) test was used to test if the data were significantly different. In contrary, if the homoscedasticity was rejected (p-value< 0.05), the Kruskal-Wallis (non-parametric) or the Kolmogorov-Smirnov (non-parametric) test with Bonferroni correction was used to test if the data were significantly different. The different letters in the graph indicate significant differences between growth conditions (13°C vs 22°C) or genotypes ($p < 0.05$). All graphs were done using ggplot2 on R except for the germination tests, bleach permeability assay and biochemical composition where Microsoft Excel was used.

References

1. M. Gou *et al.*, The MYB107 Transcription Factor Positively Regulates Suberin Biosynthesis. *Plant Physiol* **173**, 1045-1058 (2017).
2. J. Lashbrooke *et al.*, MYB107 and MYB9 Homologs Regulate Suberin Deposition in Angiosperms. *Plant Cell* **28**, 2097-2116 (2016).
3. F. Beisson, Y. Li, G. Bonaventure, M. Pollard, J. B. Ohlrogge, The acyltransferase GPAT5 is required for the synthesis of suberin in seed coat and root of Arabidopsis. *Plant Cell* **19**, 351-368 (2007).
4. Y. Li *et al.*, Identification of acyltransferases required for cutin biosynthesis and production of cutin with suberin-like monomers. *Proc Natl Acad Sci U S A* **104**, 18339-18344 (2007).
5. W. Yang *et al.*, A land-plant-specific glycerol-3-phosphate acyltransferase family in Arabidopsis: substrate specificity, sn-2 preference, and evolution. *Plant Physiol* **160**, 638-652 (2012).
6. S. Naseer *et al.*, Casparian strip diffusion barrier in Arabidopsis is made of a lignin polymer without suberin. *Proc Natl Acad Sci U S A* **109**, 10101-10106 (2012).
7. N. Rojas-Murcia *et al.*, High-order mutants reveal an essential requirement for peroxidases but not laccases in Casparian strip lignification. *Proc Natl Acad Sci U S A* **117**, 29166-29177 (2020).
8. A. L. Schilmiller *et al.*, Mutations in the cinnamate 4-hydroxylase gene impact metabolism, growth and development in Arabidopsis. *Plant J* **60**, 771-782 (2009).

9. T. G. Andersen *et al.*, Tissue-Autonomous Phenylpropanoid Production Is Essential for Establishment of Root Barriers. *Curr Biol* **31**, 965-977 e965 (2021).
10. M. Barberon *et al.*, Adaptation of Root Function by Nutrient-Induced Plasticity of Endodermal Differentiation. *Cell* **164**, 447-459 (2016).
11. J. De Giorgi *et al.*, The Arabidopsis mature endosperm promotes seedling cuticle formation via release of sulfated peptides. *Developmental cell* **56**, 3066-3081 e3065 (2021).
12. L. Demonsais, A. Utz-Pugin, S. Loubery, L. Lopez-Molina, Identification of tannic cell walls at the outer surface of the endosperm upon Arabidopsis seed coat rupture. *Plant J* 10.1111/tpj.14994 (2020).
13. J. Schindelin *et al.*, Fiji: an open-source platform for biological-image analysis. *Nature methods* **9**, 676-682 (2012).
14. P. Thevenaz, M. Unser, User-friendly semiautomated assembly of accurate image mosaics in microscopy. *Microsc Res Tech* **70**, 135-146 (2007).
15. J. De Giorgi *et al.*, An Endosperm-Associated Cuticle Is Required for Arabidopsis Seed Viability, Dormancy and Early Control of Germination. *PLoS genetics* **11**, e1005708 (2015).
16. U. Piskurewicz, L. Lopez-Molina, Isolation of genetic material from Arabidopsis seeds. *Methods Mol Biol* **773**, 151-164 (2011).
17. R. Edgar, M. Domrachev, A. E. Lash, Gene Expression Omnibus: NCBI gene expression and hybridization array data repository. *Nucleic acids research* **30**, 207-210 (2002).
18. B. Kost, P. Spielhofer, N. H. Chua, A GFP-mouse talin fusion protein labels plant actin filaments in vivo and visualizes the actin cytoskeleton in growing pollen tubes. *Plant J* **16**, 393-401 (1998).
19. L. Kalmbach *et al.*, Putative pectate lyase PLL12 and callose deposition through polar CALS7 are necessary for long-distance phloem transport in Arabidopsis. *Curr Biol* **33**, 926-939 e929 (2023).
20. S. J. Clough, A. F. Bent, Floral dip: a simplified method for Agrobacterium-mediated transformation of Arabidopsis thaliana. *Plant J* **16**, 735-743 (1998).
21. M. Iwasaki, L. Lopez-Molina, Arabidopsis thaliana Mature Endosperm Dissection and Isolation of Genomic DNA from Mature Seed Tissues. *Methods Mol Biol* **2250**, 239-243 (2021).
22. Q. Qi *et al.*, Rapid, simplified microscale quantitative analysis of lignin H/G/S composition with GC-MS in glass ampules and glass capillaries. *MethodsX* **6**, 2592-2600 (2019).
23. A. R. Robinson, S. D. Mansfield, Rapid analysis of poplar lignin monomer composition by a streamlined thioacidolysis procedure and near-infrared reflectance-based prediction modeling. *Plant J* **58**, 706-714 (2009).
24. C. M. Rolando, B.; Lapierre, C., "Thioacidolysis" in Methods in Lignin Chemistry, S. Y. D. Lin, C.W., Ed. (Springer Berlin Heidelberg, Berlin, Heidelberg, 1992), pp. 334-349.
25. F. Yue, F. Lu, R. C. Sun, J. Ralph, Syntheses of lignin-derived thioacidolysis monomers and their uses as quantitation standards. *Journal of agricultural and food chemistry* **60**, 922-928 (2012).

Supplementary Figure 1

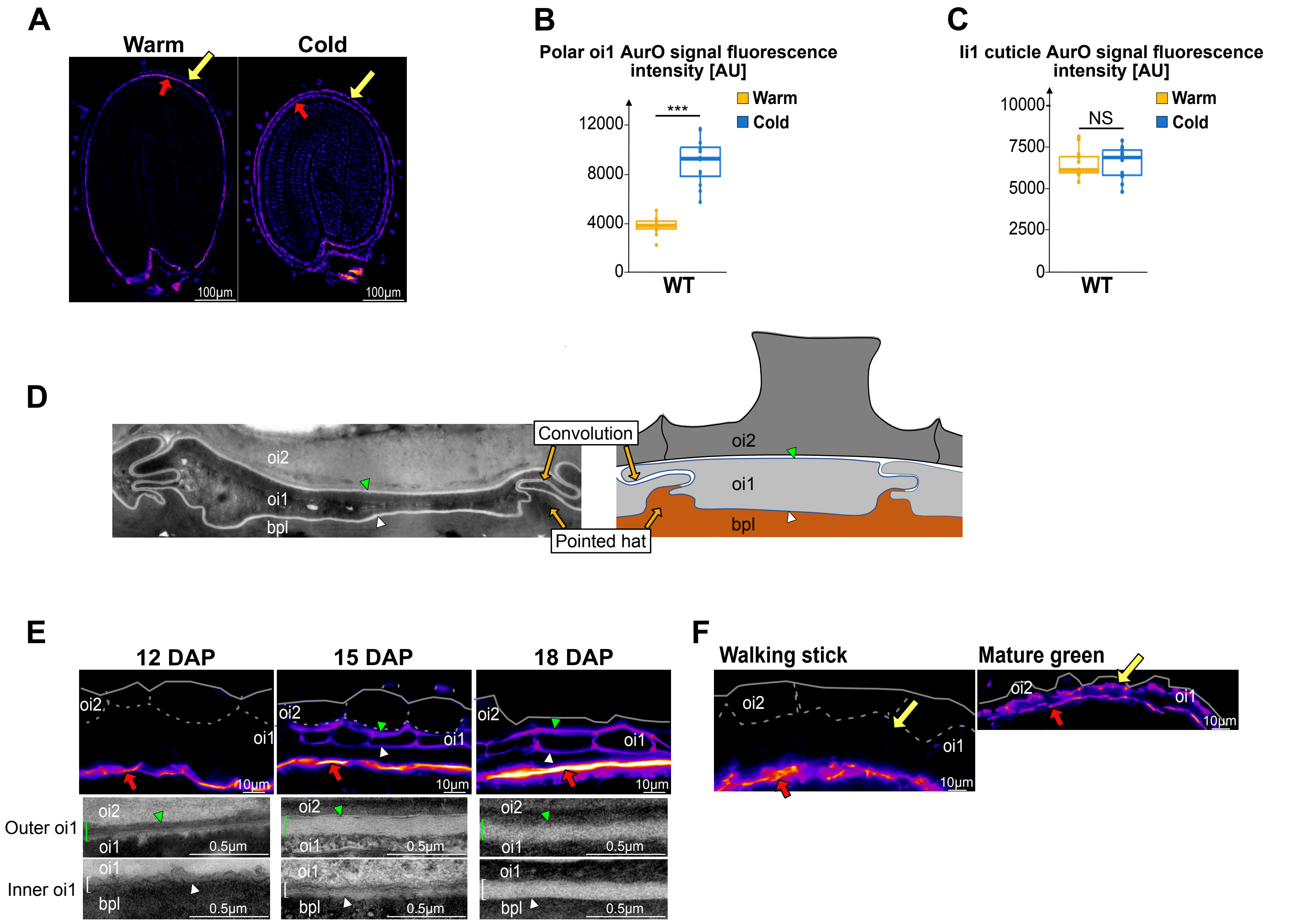

Supp. Figure 1: Identification of a polar oi1 barrier reinforced by cold during seed development

A. AurO staining of WT Warm and Cold seed sections. Yellow and red arrows indicate the AurO polar oi1 signal and the ii1 cuticule, respectively.
B. Box plots of the polar oi1 AurO signal fluorescence intensity in arbitrary units [AU]. Statistical differences assessed by Wilcoxon test (***p< 0.001, n=12 seeds per condition).
C. Box plots of the ii1 cuticle Auro signal fluorescence intensity. Statistical differences assessed by ANOVA (NS: not significant p>0.05, n=12 seeds per condition).
D. Schematic describing the prototypical convolution and "pointed hat" structure formed by the oi1 linear electro-lucent signal. TEM picture is taken from Fig.1B.
E. AurO staining and TEM micrographs showing the AurO fluorescent oi1 signal and the linear signal surrounding oi1 cells in sections of developing Warm seed at 12, 15 and 18 days after pollination (DAP), as indicated. Green arrowhead, outer oi1 barrier; white arrowhead, inner oi1 barrier, red arrow indicates ii1 cuticle, green and white brackets indicate outer and inner oi1 TEM linear signal, respectively.
F. AurO staining showing the AurO fluorescent oi1 signal in sections of developing Cold seeds (13˚C) in the walking stick and mature green stages, as indicated. Yellow arrow indicates the AurO signal from the oi1 barrier, red arrow indicates ii1 cuticle.

Supplementary Figure 2

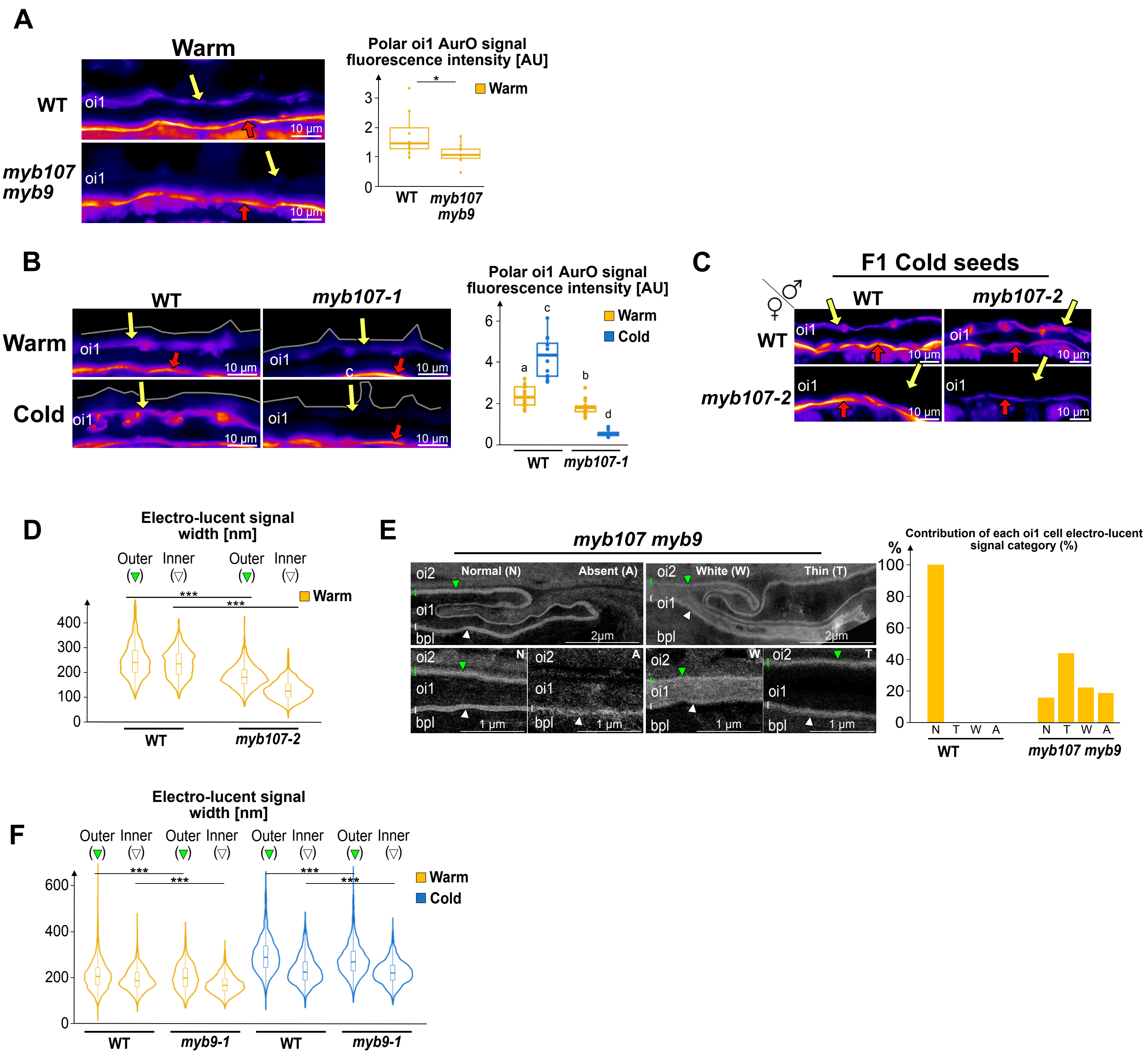

Supplementary figure 2: MYB107 is essential for polar oi1 barrier formation in seeds developing under cold temperatures

A. Left: AurO staining of WT and *myb107-2 myb9-1* (*myb107 myb9*) mature Warm seed sections. Right: Box plots of the polar oi1 AurO signal fluorescence intensity in arbitrary units [AU]. Statistical differences assessed by ANOVA test (*p< 0.05, n=12 seeds per condition). Yellow and red arrows indicate the AurO signal corresponding to the polar oi1 barrier and the ii1 cuticle, respectively.

B. Left: AurO staining of WT and *myb107-1* mature Warm seed sections, as indicated. Right: Box plots of the polar oi1 AurO signal fluorescence intensity in arbitrary units [AU]. Statistical differences assessed by Kruskal-Wallis or ANOVA test (p< 0.05, n=12 seeds per condition). Yellow and red arrows indicate the AurO signal corresponding to the polar oi1 barrier and the ii1 cuticle, respectively.

C. AurO staining of F1 Cold seeds arising from crosses between WT and *myb107-2* plants, as indicated. Yellow and red arrows indicate the AurO signal corresponding to the polar oi1 barrier and the ii1 cuticle, respectively.

D. Violin plots of the outer and inner electro-lucent signal width in WT and *myb107-2* Warm seeds shown in Fig. 2B. n=6 cells (3 seeds, 2 cells per seed), statistical analysis as assessed by Kruskal-Wallis test (***p< 0.001).

E. Left. TEM micrographs of *myb107-2 myb9-1* (*myb107 myb9*) Warm seeds showing the different categories of oi1 electron-lucent linear signal: Normal (N), Absent (A), White (W) and Thin (T). Right. Histrograms show the percentage contribution of each oi1 cell electron-lucent signal category. For each genotype, 64 oi1 cells distributed over 3 seeds were assessed.

F.Violin plots of the outer and inner electro-lucent signal width in WT and *myb9-1* Warm and Cold seeds shown in Fig. 2B. n=6 cells (3 seeds, 2 cells per seed), statistical analysis as assessed by Kruskal-Wallis test (***p< 0.001).

Supplementary Figure 3

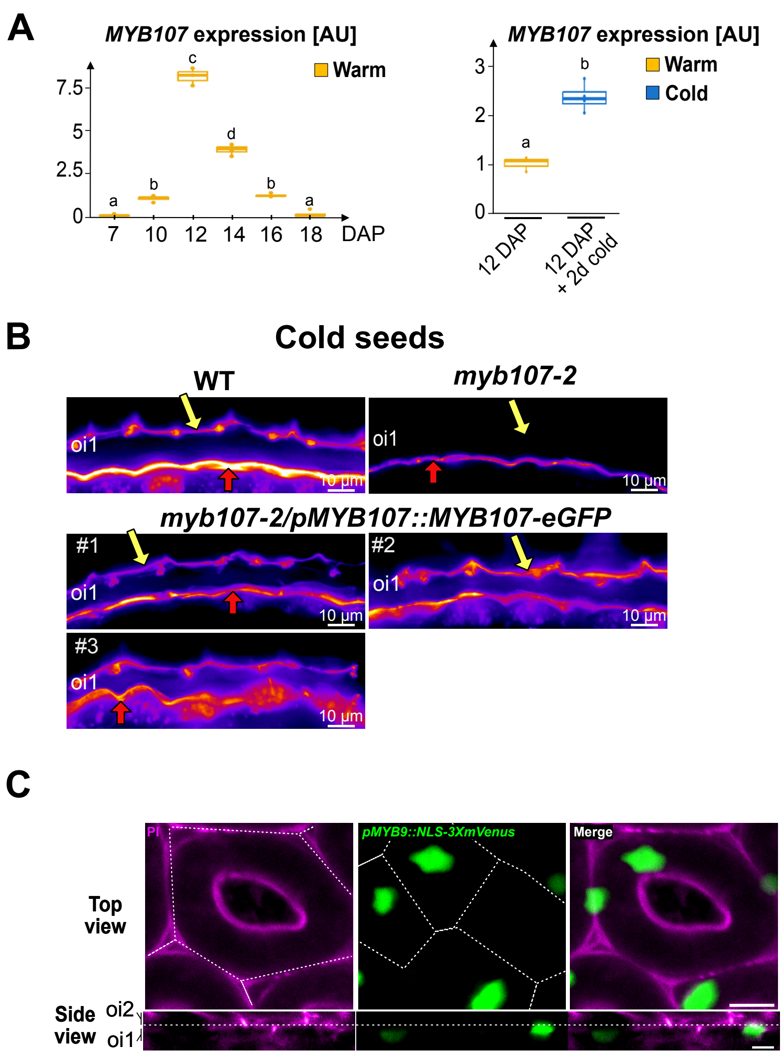

Supp. Figure 3: MYB107 is essential for polar oi1 barrier formation in seeds developing under cold temperatures

A. Left: Box plots show the relative *MYB107* mRNA accumulation WT Warm seed development at different days after pollination (DAP), as indicated. Right: Relative *MYB107* mRNA accumulation in WT Warm seeds at 12 DAP and after a two days upon transfer to cold. Expression levels were normalized to those of CDK2. n=3-4 technical replicates. Statistically significant differences between the different conditions are indicated by different letters as assessed by a Kruskal-Wallis or ANOVA test ($p < 0.05$).

B. AurO staining of WT, *myb107-2* and *myb107-2/pMYB107::MYB107-eGFP* Cold seed sections, as indicated. Yellow and red arrows indicate the AurO signal corresponding to the polar oi1 barrier and the ii1 cuticle, respectively. Three independent complementation lines are shown (#1, #2 and #3).

C. Confocal images showing propidium iodide (PI) and GFP fluorescence in the seed mature green stage of WT/*pMYB9:NLS-3XVenus* transgenic plants. Bar, 10 µm.

Supplementary Figure 4

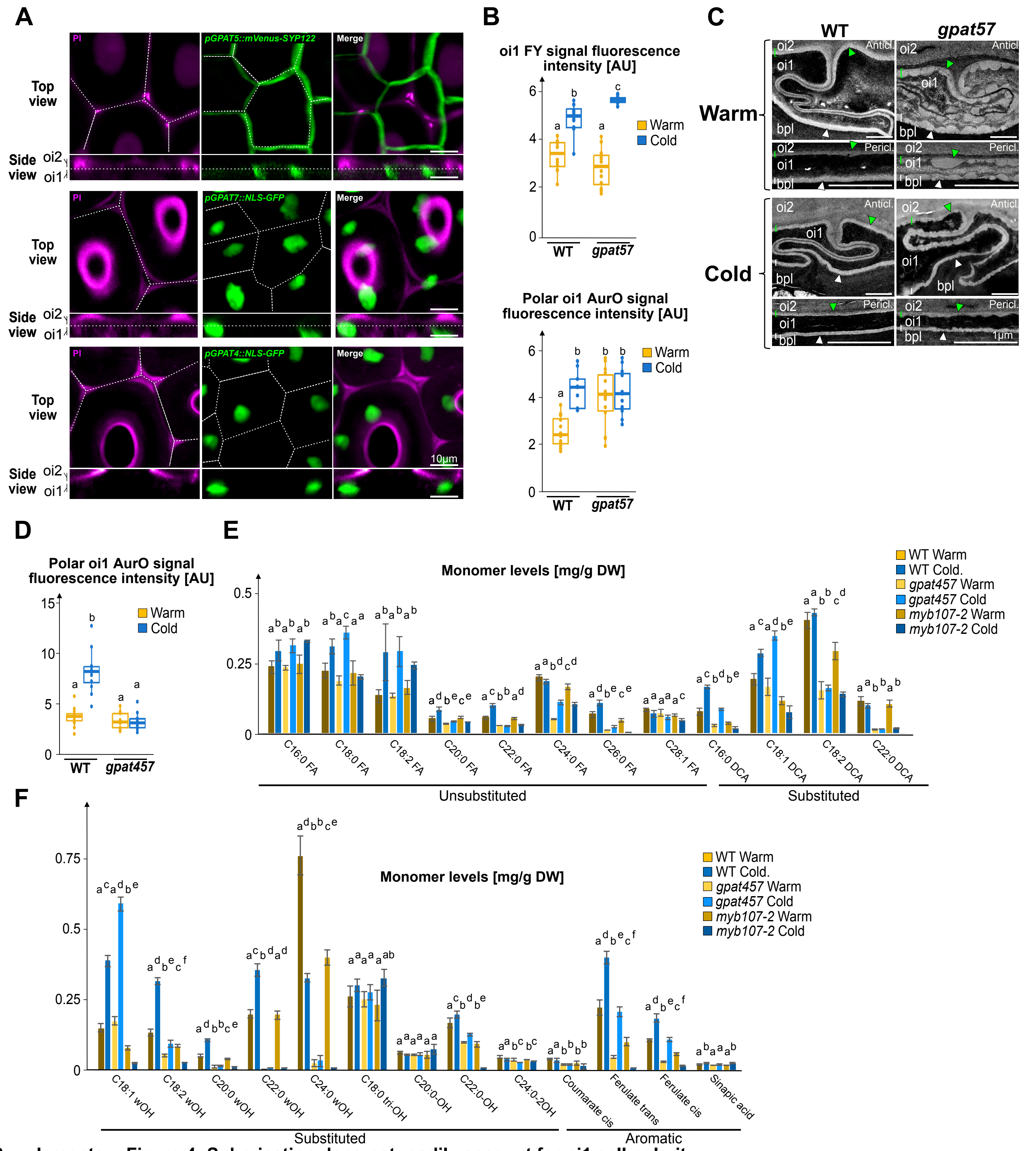

Supplementary Figure 4: Suberisation does not readily account for oi1 cell polarity

A. Confocal images showing propidium iodide (PI) and GFP or Venus fluorescence in the seed mature green stage of *pGPAT5::mVenus-SYP12*, *pGPAT7::NLS-GFP* and *pGPAT4::NLS-GFP* transgenic plants, as indicated. Bar, 10 µm.

B. Top: Box plots of the oi1 FY signal fluorescence intensity in Fig. 3B. Statistically significant differences between the different conditions are indicated by different letters as assessed by a Kruskal-Wallis or ANOVA test ($p < 0.05$, n=12-13 seeds per condition). Botton: Box plots of the polar oi1 AurO signal fluorescence intensity in Fig. 3B. Statistically significant differences between the different conditions are indicated by different letters as assessed by an ANOVA test ($p < 0.05$, n=12-14 seeds per condition).

C. TEM micrographs showing the linear electron-lucent anticlinal (Anticl.) and periclinal (Pericl.) signal surrounding oi1 cells in WT and *gpa57* Warm (W) and Cold seeds, as indicated. Green and white brackets/arrowheads indicate the outer and inner oi1 electro-lucent signal, respectively.

D. Box plots of the polar oi1 AurO signal fluorescence intensity in WT and *gpat457* Warm and Cold seeds, as indicated. Statistically significant differences between the different conditions are indicated by different letters as assessed by an ANOVA test ($p < 0.05$, n=11-12 seeds per condition).

E and **F:** Polyester monomer levels in WT, *gpat457* and *myb107-2* mature Warm and Cold seeds, as indicated. Values represent means ± SD, n = 4. For each monomer, different lowercase letters indicate significant differences, as determined by Krusal-Wallis or one-way ANOVA test, $p < 0.05$

Supplementary Figure 5

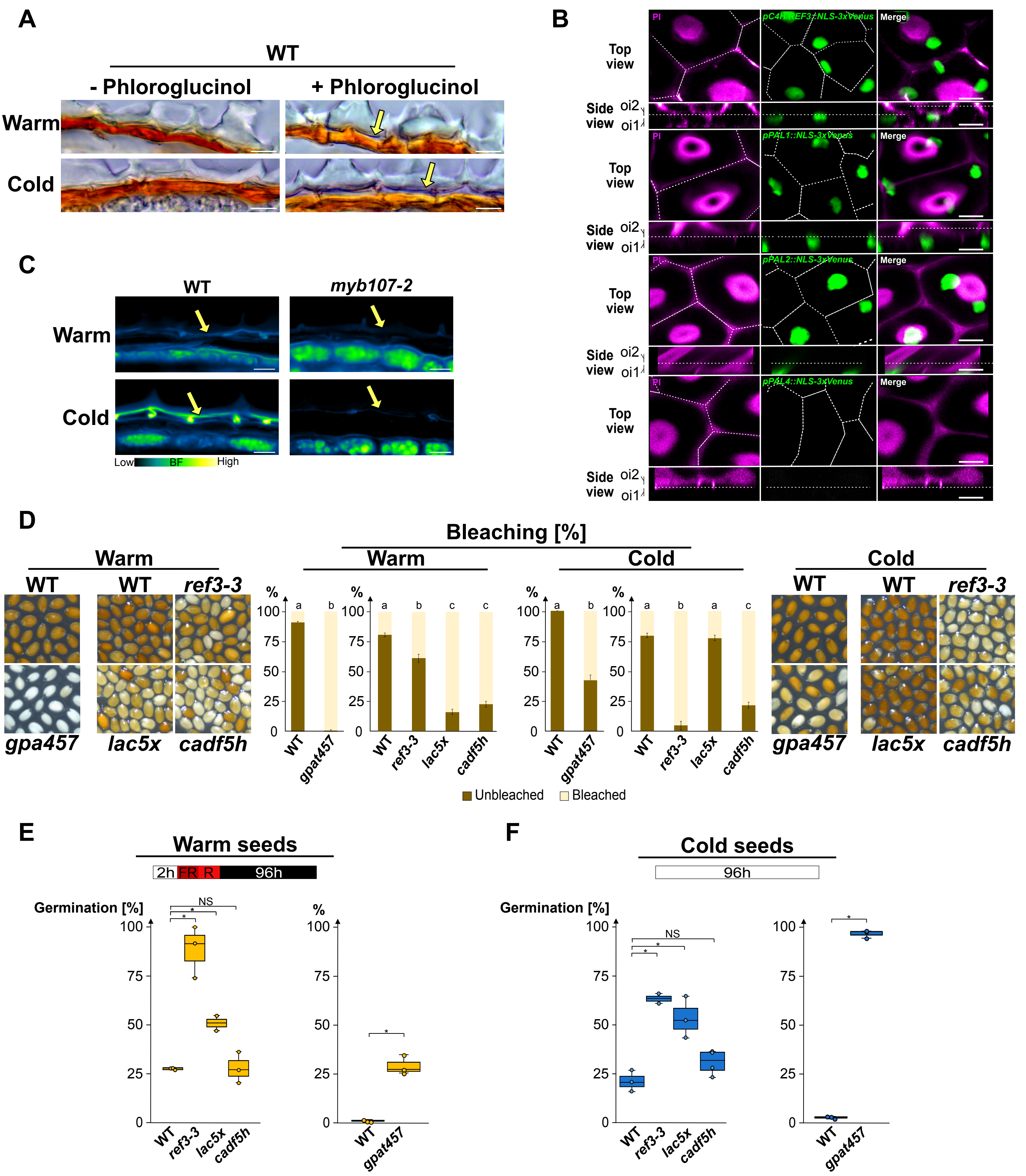

Supplementary Figure 5: Cold promotes the polar deposition of lignin or lignin-like polymers in oi1 cells

A. Pholoroglucinol staining of histological sections of WT mature Warm and Cold seeds. Yellow arrow indicates a purple signal between the oi1 and oi2 cells that is more visible in Cold seeds. Bar, 10µm.

B. Confocal images showing propidium iodide (PI) and Venus fluorescence in the seed mature green stage of *pC4H/REF3::NLS-3xVenus*, *pPAL1::NLS-3xVenus*, *pPAL2::NLS-3xVenus* and *pPAL4::NLS-3xVenus* transgenic plants, as indicated. Bar, 10 µm.

C. Basic Fuchsin (BF) staining of WT and *myb107-2* mature Warm and Cold seed sections. Yellow arrow indicates the BF signal corresponding to the polar oi1 barrier. Bar, 10µm.

D. Same experiment and statistical analysis as in Fig. 5A except that *gpat457* seeds were incubated for 2h in 1.5% sodium hypochlorite and *ref3-3*, *lac5x* and *cadf5h* Warm and Cold seeds were incubated for 2h and 3.5h in 2.5% sodium hypochlorite.

E. Box plots showing germination percentages of freshly harvested Warm mature seeds of various genotypes, as indicated. Statistics as in Fig. 5A

F. Same as E using WT and *ref3-3*, *lac5x* and *cadf5h* seed one week after harvest and WT and *gpat457* seeds 36 days after harvest. Statistics as in Fig. 5A.
